# Diagnostic testing: therapeutic mobilities, social fields, and medical encounters in the transnational healthcare practices of Polish migrants in the UK

**DOI:** 10.1016/j.jmh.2022.100100

**Published:** 2022-04-05

**Authors:** Giuseppe Troccoli, Chris Moreh, Derek McGhee, Athina Vlachantoni

**Affiliations:** a Centre for Research on Ageing and Centre for Population Change, University of Southampton, Southampton SO17 1BJ, United Kingdom; b School of Geography, Politics and Sociology. Newcastle University, Newcastle upon Tyne NE1 7RU, United Kingdom; c Faculty of Social Sciences, Keele university, Newcastle upon Tyne ST5 5BG, United Kingdom

**Keywords:** Transnational healthcare, Therapeutic mobilities, Tests, Transnational social fields, Polish migration

## Abstract

While diagnostic tests are a fundamental component of contemporary medical practice they are seldom considered in studies of transnational healthcare. This article investigates the little-studied role played by diagnostic testing in the healthcare-seeking practices of migrants. It is concerned with the experiences of Polish migrants living in the UK and who access a variety of health services in their host and origin countries across the public and private sectors. We analyse data from semi-structured phone interviews conducted in 2020 with 32 adult Poles living in the UK who identified as having themselves, or non-professionally caring for someone with, a long-term health condition. The article contributes to the literature on migrants’ transnational healthcare practices by showing the centrality of diagnostic technology in their health management and sense-making through the creation, modification, and maintenance of ‘transnational social fields’ (Levitt and Schiller 2004). By emphasizing the role of tests in the patient-doctor relationship the article exposes the therapeutic outcomes of the mobilities of patients and tests as they intersect with physicians in multiple medical encounters.

## Highlights

• Diagnostic testing is seldom explored in studies of migrants’ healthcare practices.
• Tests’ availability and doctors’ willingness to discuss results are crucial for migrants’ evaluation of the care received.
• Test results are moved between private and public sectors and national borders.
• Movements of patients and tests establishes relationships with doctors and have therapeutic effects.
• Future studies should consider testing as a fundamental component of migrants’ health.

## 1 Introduction

This article focuses on the role played by diagnostic technology in migrants’ pursuit of healthcare. Through exploring the experiences of Polish migrants living in the UK, the article exposes the relevance of diagnostic tests as an overlooked enabler of transnational healthcare. The transnational paradigm has brought to the fore links between people, places, and institutions across nation-states (Vertovec, 1999). Within the study of healthcare, scholars argue that transnationalism has the potential of at once considering the possibilities and limitations available in localities and the connections between them (Zanini et al., 2013). Increasingly, the healthcare seeking of migrants has been studied as a transnational practice (Villa-Torres et al., 2017). A number of studies have pointed to the role of social relationships in transnational healthcare practices (Goodwin et al., 2012, Bochaton, 2015). Levitt and Glick-Shiller (Levitt and Glick-Schiller, 2004) have argued for the incorporation of migrants within ‘transnational social fields’, ‘as a set of multiple interlocking networks of social relationships through which ideas, practices, and resources are unequally exchanged, organized, and transformed’ (Ibid. pg. 1009) between actors located across national borders. Social fields, they argue, have fluid boundaries, contain experiences, organizations and institutions, and are created by their participants who can simultaneously engage in a multiplicity of transnational processes.

To emphasize the relational aspect of these practices we situate healthcare seeking within social fields that span national borders and follow migrants in their creation and navigation of these fields. With this premise, we build on the literature on ‘therapeutic mobilities’ (Kaspar et al., 2019) applying a mobility lens that poses the multiplicity of things and beings in motion related to health. While the therapeutic can be an outcome of the act of moving (Gatrell, 2013), it is also generated from the encounter of various mobile subjects in relation to each other and is transformative of them (Kaspar et al., 2019). Thus, we see migrants-patients partaking in different mobilities transforming themselves and the social fields they are part of. Further, while the scholarship on migrants’ transnational healthcare practices has a primary focus on people and relationships and to a lesser extent on things (Villa-Torres et al., 2017), therapeutic mobilities facilitate the exploration of the multiplicity of things and beings in motion related to health, thus extending the analysis to the mobility of pharmaceuticals (Bochaton, 2019) and other products (Walton-Roberts, 2019). We contribute to this literature by exploring the mobilities associated with an under-researched enabler of transnationalism: diagnostic tests.

Within the study of transnational healthcare, medical travel and medical tourism, diagnostic tests are not considered a crucial element of healthcare seeking mobilities. Medical check-ups are one of the services targeted by medical travellers (Chee et al., 2019). Migrants can find their expectations to undergo diagnostic procedures in their country of residence unmet (Raffaetà, 2019), and as a consequence travel to satisfy these expectations (Bradby et al., 2020) or to access testing and its outcomes more swiftly (Lee et al., 2010). We know that patients who are transnationally mobile can access diagnostic testing, and that this is an outcome of their expectations, needs and dependent on the availability of diagnostic testing, however, what tests do, what patients do with tests or use them for, and how they think of and through tests is an overlooked aspect of transnational healthcare research. This is perhaps surprising given the relevance of tests in understanding one's health and in healthcare seeking. Diagnostic tests are one of the ways in which information is gathered in order to reduce uncertainty in therapeutic decisions (Balogh et al., 2015). While tests do not necessarily lead to greater certainty, they are pivotal to the management of uncertainty about one's condition by increasing accuracy in diagnosis (Marcum, 2008). Diagnostic tests have a central role to play before a disease is suspected, when there are symptoms, and after a condition is diagnosed. Test results are “instrumental” (Leder, 1990 pg. 15) or “technological texts” (Jutel, 2011 pg. 126), which are interpreted by doctors. When they explain the meaning of test results to their patients, clinicians make them part of the testing process and allow patients to be part of the interpretative process as active participants (Marcum, 2008 pg. 86).

In this paper, we examine how tests are at once detachable from patients and used by them to establish social relations transnationally through multiple medical encounters with physicians. Tests provide information to patients about their own bodies through the means of professional medical interpretation of them. The inclusion of patients in the interpretation of results happens usually in clinical encounters between patients and doctors. There, patients’ subjective narratives are transformed by doctors who legitimize them as medical narratives: the two might align, when a patient sees their lived experience as recognized and validated by a diagnostic interpretation, or alternatively, when patients find that the interpretation of diagnostic tests do not explain their symptoms and experiences (Jutel, 2009). Relationships in clinical encounters are characterized by both patients resistance to physicians’ power when their narratives do not align, and alliance with doctors to restore their functions (Filc, 2006). Test results are the epitome of disembodied clinical evidence, and their relevance in the clinical encounter is mainly given by their reading by the clinician (Leder, 1990). We show how patients act on the relations with physicians and the resulting therapies by a series of mobilities in which they simultaneously exploit their disembodiment and seek to include physicians in their care.

Tests mediate patients' understanding of themselves relationally, through doctors. We situate the centrality of medical encounters in migrants’ social fields. Our approach in marrying a transnational social field approach with a therapeutic mobilities lens has enabled us to examine the social relationship of the patient-doctor dyad and its actualization during care encounters when patients, tests and clinicians come together. Thus, by immersing the patient-doctor relationship in a transnational context we expose how migrants act upon their health situation by multiplying and modifying these encounters during which their therapeutic outcomes are relationally produced. We argue that diagnostic tests are often the resultant artifact of these encounters that patients can take away with them in the furtherance of their healthcare seeking across sectors and across national boundaries. During clinical encounters patients and their tests circulate between and amongst a diverse range of clinicians in different healthcare systems and in different countries.

Approaches to transnational healthcare focusing on space have highlighted the development of multiple socialites at specific medical localities (Chee et al., 2019). Medical travel destinations are constituted during care encounters between patients and doctors when the sets of relations that connect different entities are activated (Kaspar and Reddy, 2017). This follows an emphasis on the fact that transnationalism takes place in specific spaces and times. Connectivity between mobile and immobile agents intensifies during their bodily co-presence (Pordié, 2013). At the same time, patients move with ‘mobile prosthetics’ (Ormond, 2015, Bissell, 2009) which make their medical travels possible. We focus on the medical encounters to show how they actualize connections between patients, the tests they seek and carry with them, and practitioners. Thus, instead of centring on space, our analytical focus is on the mobilities of diagnostic test carrying patients and how their healthcare seeking aims are enabled (or not) during clinical encounters with different physicians in different places. Rather than stressing the multiple socialites as they overlap, we focus on the multiplicity of mobilities of patients and tests and their role in how the social field is built, maintained, and modified. Thus, our analysis of intersecting mobilities centres on the social aspect of the patient-clinician dyad, which is part of the social field and is activated during medical encounters. Partially, this is an outcome of our methodological focus on U.K. resident Polish migrants’ experiences of seeking healthcare and reaching towards a variety of different health service providers variously located in different countries. While for our purposes this focus deemphasizes other types of mobilities (including mobile healthcare professionals) and other socialites involved in transnational healthcare, it allows an analysis that appreciates how the therapeutic effects of the mobilities of patients and tests are entangled with the relationalities emerging from the specific qualities of tests as they are concretized during clinical encounters which inform care trajectories. The significance of tests in these encounters comes from their role in confirming and defining the cause of suffering in the therapeutic management of illness.

In the next section, we contextualize the healthcare practices of Polish migrants living in the U.K. and specify the methodology used in the study on which this article is based. It follows the presentations of results in which we draw from participants’ experiences to expose the meanings, relationalities and mobilities involved in diagnostic procedures. Finally, we discuss our findings to highlight the relevance of tests and the potential of studying transnational healthcare focusing on the mobilities of multiple entities and experiences as they connect during medical encounters.

### 1.1 Transnational healthcare practices of Poles in the UK

An increase in the flow of patients from the U.K. to Poland since the mid-2000s is mostly constituted of migrants seeking to access healthcare in their home country (Horsfall, 2020). Returning to the country of origin to access medical services is common to other Europeans living in the U.K. (Moreh, McGhee, and Vlachantoni, 2018) and has been found for Poles in several of EU countries (Mathijsen, 2019, Migge and Gilmartin, 2011, Main, 2014). By moving from Poland to the U.K., Poles shift between state-funded health systems and lose access to public services in Poland, while becoming entitled to the use of the National Health Service (NHS) in the U.K.. Poles access most of the services in Poland within the private healthcare sector (Troccoli et al., 2021), thus they seek healthcare “on their own initiative” outside the EU institutional routes (Legido-Quigley et al., 2011). This reflects both the loss of entitlements to public healthcare and the presence of a developed private healthcare market in Poland to which Poles are habituated, not least for its degree of integration with the public system (Sowada et al., 2019).

Alongside medical consultations and therapies during their travels to Poland (Sime, 2014, Main, 2014, Smoleń, 2013), Poles access Polish clinics in the UK catering to the migrant population (Bell et al., 2019, Lindenmeyer et al., 2016, Main, 2016). The literature exploring the healthcare of Polish migrants in various UK localities mentions GP reluctance to perform extensive tests as one of the elements judged critically by Polish migrants (Goodwin et al., 2012), alongside more frequent screening in Poland for specific conditions (Jackowska et al., 2012, Main, 2014, Crowther and Lau, 2019), and UK GPs’ prescribing practices, therapeutic recommendations, perceived lack of competence, short consultation times, reluctance to physically examine patients and refer them to specialist services (Madden et al., 2017). Such differences partly reflect the extent to which expectations are met when differences in medical approaches and protocols between countries emerge. However, beyond the lamented absence of diagnostics and the fact that they are one of the services sought transnationally, the particular gap in knowledge that this paper explores is what patients actually do with tests and what tests enable in terms of their transnational health seeking practices.

## 2 Methodology

This article is based on data collected in the qualitative phase of a mixed-methods study aimed at gathering primary data at the UK level on how Poles manage their and their families’ healthcare transnationally. Ethical approval was granted by the Research Integrity and Governance team (52861, 52861.A1) at the University of Southampton. In order to maintain participants’ anonymity and confidentiality we assigned a (unique) pseudonym to each of them and omitted any identifying detail from the results that follow. The study design was sequential (Creswell, 2015), with semi-structured telephone interviews being conducted with participants selected from among those who responded to an online nonprobability survey.

Through single and multiple-choice answers and open-ended responses, the online survey gathered information on healthcare access, and demographic and health-related characteristics between November 2019 and February 2020. Respondents (N 510) were recruited through advertisements on news websites and Facebook groups for Poles in the UK, and via direct e-mail to participants in a previous study. To be eligible to participate in the study, respondents had to be over eighteen years old, be Polish citizens – or children of a Polish citizen – and living or having lived in the UK and have consulted a medical professional in the UK.

The second phase of the study consisted of semi-structured interviews with a purposeful sample drawn from the survey respondents who agreed to take part in further research and identified as suffering from a long-term health condition or caring in a non-professional capacity for someone who is chronically ill. This ensured that participants were more likely to have enduring experiences with healthcare services while keeping a multiplicity of perspectives grounded in the variety in number and severity of the health conditions present in our sample. Participants were invited to take part via an email with details of the study and information about the interview, including the offer of a small coupon as a gratitude gesture. Informed consent was obtained via email.

Each participant (N 32) was interviewed once between June and August 2020 by the lead author. All participants were living in the UK at the time of the interviews (mean 10 years), they were aged between 20 and 63, 22 were female, 26 had a long-term condition and 10 cared for someone with a chronic illness (see Table 1). The preliminary analysis of the data from the first phase of the research informed the choice of themes that were explored during the interviews. Participants were chiefly asked to recount their experiences related to their or their families’ health conditions, in addition they were also asked to elaborate on their plans and to evaluate and compare their past experiences (see Appendix). Data collection was ended after the opinions and experiences expressed in the interviews became repetitive, that is saturation was reached and interviewees were not bringing up new themes relevant to the research objectives. All interviews were conducted remotely, either via phone or via computer software (audio-only) and were recorded. The interviews lasted an average of 64 minutes and were conducted in Polish, except for one which was in English following the preference of a participant. They were successively translated and transcribed into English and the translation was validated by the lead author.

**Table 1:** Participant demographic characteristics.

N = 32		N
Age		
	20-29	2
	30-39	10
	40-49	11
	50-59	6
	60-69	3
Sex		
	Female	22
	Male	10
Has a long-term condition/chronic illness		
	Yes	26
	No	6
Was diagnosed with a chronic condition before moving to the UK^a^		
	Yes	12
	No	14
Cares for someone with a long-term condition/disability		
	Yes	10
	No	22
Years spent in the UK at the time of the interview		
	1-4	4
	5-9	11
	10-14	14
	15-16	3
Household monthly income (£)		
	less than 956	1
	956-1283	6
	1283-1591	4
	1591-1922	5
	1922-2289	1
	2289-2735	2
	2735-3264	2
	3264-3986	4
	3986-5273	6
	5273 or more	1

a Excluding participants who do not have a long-term condition.

In analysing the data, we used an inductive approach in which a first scrutiny of the data led to identifying emergent themes and successively developing codes to frame the material. Although participants were not asked specifically about tests, the prominence of diagnostics as a recurring theme in the open questions of the survey led to the development of thematic coding which was then applied to the interviews. Two codes were created to aid the analysis of the interview transcripts. One code highlighted interviewees’ mentions of diagnostic procedures such as tests and scans. Another code highlighted the circumstances of participants’ diagnoses, including the acceptance in the UK of diagnoses made in Poland and the diagnostic processes which had not yet led to a diagnosis. The analysis preceded by comparing the coded segments between each other (that is between participants) and iteratively situating each participants’ engagement with diagnostics within their history of healthcare service use (that is within each transcript). This allowed to identify three main themes of analytical relevance by which we divide our results.

## 3 Results

We present our results in the three sections that follow. First, we address the meaningfulness of diagnostic procedures for our participants and the relationalities that are enabled by them (3.1). Based on patients’ depictions of encounters with medical professionals, this section shows how diagnostic procedures enabled an understanding of both participants’ own health and the care they received, and the importance of the interactions prompted by test results for the doctor-patient relationship. Second, we expose the mobilities of patients to be tested and the asymmetries which they entail (3.2). Drawing on migrants’ accounts of seeking to undergo diagnostic procedures, this session focuses on their movement within and between the public and private sectors in the UK and Poland, the relations these movements entail, and the unevenness which characterizes them. Third, we follow the mobilities of tests and their therapeutic effects (3.3). By employing patients’ descriptions of how they submitted test results to multiple scrutiny this section shows the impact of the mobilities of test results on migrants’ medical treatments and health through the consolidation and formation of social relationships with healthcare professionals.

### 3.1 Care as tests, testing as enabling care

Doctors’ willingness to refer for testing was a key feature of most participants’ discussion of the care they had received. Participants expected practitioners to refer them to diagnostic exams to further understand why they were experiencing symptoms and asked them to do so when they seemed unwilling. When their illness was known to them, participants understood tests as necessary to further understand its origin and manage its progression.

Karolina recalls when a personally stressful time and demanding job were coupled with feeling constantly unwell. She went to her General Practitioner (GP) in the UK:

I explained how I felt and what my symptoms were. I was referred for tests, which I did the same day or day after. Later, my doctor, a wonderful man, always interested in his patients and their complaints, always prepared, explained everything to me regarding what was happening with my thyroid and what could be done. He suggested that I start taking medication, which I did and I started feeling better.

[Karolina, F, late-40s]

In Karolina's portrait of her GP, referral for tests characterizes his practice in highly positive ways. Conversely, most participants judged the care received as insufficient when they felt health practitioners’ attitudes in the UK were dismissive. That is, their symptoms and pain were not investigated by the means of diagnostic technology and they were prescribed medications that superficially addressed their sickness. Recently Karolina's overall condition worsened, and new symptoms and pains surfaced. Further scans prescribed did not highlight anomalies, and she was prescribed by another doctor at her GP surgery a medication that was provoking unpleasant side effects:

I feel like I've been dismissed there, to put it mildly… Basically, what she was saying was ‘keep taking the tablets and if there's no improvement after three months, call me’. And that's really it in terms of tests… I can't do much more than that. […] my doctor isn't really that interested in analysing my health issues too deeply with me.

[Karolina, F, late-40s]

Disappointment similarly arose when patients' own specific requests for being tested were disregarded by physicians. Iwona experienced this refusal:

I told him [the GP] that I was feeling really fatigued and lethargic, but he said it was to do with stress, and that he couldn't see any reason to refer me for tests, and he didn't; instead, he told me to get some multivitamins.

[Iwona, F, mid-40s]

Even when a diagnosis was explicated, and a treatment prescribed care was judged as superficial if it avoided looking for the aetiology of the disease by the means of further tests. Mikołaj could not explain to himself the reason behind his hypertension:

They just gave me some tablets and checked how I responded to them, but the truth is that I still don't know the reason for my high blood pressure. It might be genetic, because my mum and gran both had high blood pressure. […]. Nobody is doing anything to find out… I understand there are a lot of patients, but it seems that that's how they do it here - they give you medication and ’it's all fine, go home, go to work’…

[Mikołaj, M, late-40s]

Tests were needed at once to understand one's health and how well it was managed by clinicians. Interviewees’ feelings of being taken into consideration and informed about their health were related not only to the presence or absence of tests but also to the extent to which the results of the tests mediated their understanding of their health. Several respondents who articulated their care in terms of tests placed importance on the fact that the discussion of the outcome of diagnostics prompted them to meet the doctor who through a thorough explanation of the results allowed them to comprehend what was happening to them. Therefore, what was important for participants was not only undergoing tests, but also accessing their results as Emilia describes:

My husband had a full blood count tests twice; the doctor called and said that the results were a little bit better than last time, and that was it… The second time nobody even called to say what the results were… You don't get a copy of the results here… In Poland you get a copy of the results and you show to your doctor… The doctor then talks you through your results and explains what is within the norm and what is not, and tells you whether there's any reason for concern.

[Emilia, F, late-50s]

Thus, tests mediated participants’ understanding of their health by being ‘read’ to them by practitioners. Test entailed a relationship with practitioners who referred to and explained results. The interpretation of test results during medical encounters positively consolidated the patient-doctor therapeutic relationship.

### 3.2 Moving to be tested

Participants who were determined to be tested for the purpose of diagnosing the cause of ill-health, to maintain their health status or manage a known and ongoing condition were often mobile within the NHS and also to the private health care sector to access diagnostics. Within the NHS, participants requested that their GP facilitated their access to specialists. Most of our respondents also accessed healthcare outside of the NHS. The majority accessed the Polish private sector, often combining testing and specialist consultation with regular visits. Less often, migrants accessed private services in the UK, mostly offered by Polish-staffed clinics. These movements within and between sectors entailed different degrees of physical movement and were often enacted by the same participant.

Their insistence on receiving care which included the use of diagnostic testing included a few examples where they changed health professionals within the NHS. Primarily, they obtained the level of care that they deemed appropriate through gaining access to doctors who enabled them to be tested and achieve a diagnosis. For example, Paulina was referred by her GP to an otolaryngologist:

The doctor I saw completely ignored me. I went to see him after I'd lost hearing in the right ear and something started happening on the left side as well. I suffer from really bad ringing in my ears and have a problem with communication… […] The doctor I'd seen didn't do any tests, nothing…

[Paulina, F, mid-30s]

Paulina asked to be referred to a different specialist and this time she met a doctor who showed interest:

They took my medical history and carried out comprehensive tests, and referred to me to a hospital in London, to a really good ENT [i.e. internal medicine] doctor there, who did a whole range of tests. I had everything done: CT, MRI, brain tests, everything… and then everything became clear.

[Paulina, F, mid-30s]

Paulina's disappointment with the absence of medical tests in the care she received prompted her to obtain access to be seen within the NHS by a different specialist who thoroughly tested her and clarified to her the causes of her hearing issues. While waiting for this change, she underwent testing in Poland to have quick reassurance about her health condition. Moreover, since she is also affected by a thyroid disfunction, she repeated some of the tests she underwent within the NHS because she had become aware of the different ways of testing between countries:

I suffer with thyroid problems and other health issues, all genetic problems, so once a year I do some tests in Poland, because they have different norms for thyroid function there. I know of people who are diagnosed in Poland with thyroid problems, but in the UK their results come back as are normal. […] Once a year I request a general health check from my doctor, but because I don't completely trust the results, when I go on holidays to Poland, I repeat those tests there, and then I know whether the tests from the UK are true or not, and so far that's been ok.

[Paulina, F, mid-30s]

Paulina's movements within and between sectors show how access to testing can be multiple and respond to various needs. By duplicating or repeating tests privately participants sought reassurance in terms of reaching out to more than one diagnostic system and practitioner. That is, while tests were themselves a commodity sought transnationally for their intrinsic differences, they were also a means to access various doctors whose attitudes and opinions were compared. Some test results were intelligible to patients, who could have unmediated access to information about their health when they sought to be screened beyond what their general practice doctors prescribed. However, even patients who were confident about their medical competence did not exclude the professional interpretation of results when they independently accessed diagnostics. Agata maintains her health by being regularly checked via tests purchased in Poland which she can read herself. However, this does not mean the exclusion of her GP in the UK:

I get all tests done in Poland; the only thing I do in the UK is when I go to my GP - for whatever reason - I show them tests’ results and ask to add them to my file. I explain a little bit about the results. Blood test results look pretty much the same [in Poland and the UK]; the only thing is that addresses and information are in Polish, but generally they state a range, a level of something, showing clearly whether they are normal or not, and I am able to read them. I regularly update my doctors here…

[Agata, F, early-30s]

The need to monitor and investigate their health status prompted participants to move within and beyond the NHS to reach a level of care they deemed satisfactory. Even when pursuing tests without the mediation of a doctor prescribing them, tests were eventually submitted to the attention of clinicians. When moving to be tested, within or outside the NHS, participants also widened and modified the relationships they had with medical professionals.

When participants accessed tests that were not prescribed by them within the NHS, or consulted professionals not referred by the GP they usually did so by paying for tests in the private sector. Private services in the UK were commented upon as particularly expensive and often out-of-reach and were used by a minority of participants. Moreover, Polish clinics in the UK are concentrated particularly in London, which makes them comparatively less accessible for participants living elsewhere in the country. More commonly, private healthcare was accessed in Poland where it is considered to be much more affordable also because of the difference in purchasing power given the higher labour and money's value in the UK, as Iwona explains:

It's great to go to Poland if you live and earn money here. When I go on holidays to Poland and have a couple of thousand in my wallet, I can go to any clinic I want, wait a quarter of an hour, go in and have a scan done… I haven't got higher education and am an unskilled worker effectively, so if I lived in Poland, I wouldn't be able to afford having tests done privately.

[Iwona, F, mid-40s]

However, this does not mean that tests were available to all. Mikołaj reflects on the obstacles to thoroughly investigate his underlying health conditions:

I would have to get some tests done and I would have to do that privately to have it done relatively quickly. That requires money and time… I would have to stay there for at least three weeks to do it thoroughly. […] on the one hand, I would like to do that, on the other hand, I don't have the funds for it at the moment; it obviously costs to do it privately. I think I would have it done in Poland, because it's cheaper.

[Mikołaj, M, late-40s]

The mobilities entailed in the access to private care are contingent upon financial ability to purchase healthcare services, the time to travel to Poland and depending on the condition the availability of time to undergo the diagnostic tests required. In curtailing the possibility of being tested, these elements also detract from patients’ capacity to actively seek understanding of their health and the impact the therapeutic possibilities which are enabled through the relational potential of tests.

### 3.3 Moving (with) tests

While visiting Poland, Agata noticed a lump on her throat and underwent a scan there which highlighted the presence of nodules. In the UK, she was prescribed a biopsy with the NHS:

I had a biopsy done in the UK, which I had to wait a month for. I had it done again in Poland, just because when you use private health service in Poland, you just make a call and within 48 hours you get an appointment with a specialist, and that appointment also included a biopsy and scan. I have been tested both in the UK and in Poland.

[Agata, F, early-30s]

Agata widened her social field in relation to her undiagnosed condition by being tested both within and outside the NHS and consulting different professionals at the point of testing. Having test results at hand allowed her to receive different opinions about her condition in the UK. One NHS specialist doctor suggested surgery but given the apparent young age of the professional and the long-term implications of the procedure, she decided not to be operated on. She consulted another doctor, described as older, who instead advised not to act if the situation remained unchanged and no discomfort emerged. Further, her homeopath in Poland agreed with this course of action. Thus, after several tests did not show anything abnormal, and the medical opinions she received from a trusted professional assured her that there was no need for treatment, Agata was reassured and stopped seeking a diagnosis. Thus, by moving tests and encountering multiple professionals Agata was able to have the confidence to diverge from the initial therapeutic recommendation and did not undergo a major surgery through initiating a corroborative triangulation of opinions across national boundaries, sectors and healthcare traditions.

While to be tested one needs to move, the portability of tests means that patients can move with them between practitioners and sectors, and thus submit the same diagnostic evidence to multiple scrutiny. While tests results are disembodied from patients which might lead to the depersonalization of the clinical encounter (Leder, 1990), by moving (with) tests our participants actively established relationships with medical professionals, or actualized established ones, in clinical encounters which impacted their health. The insistence on having test results at hand signals the importance of having understanding mediated through tests by doctors and the importance of being able to multiply opinions given the detachable quality of test results.

By moving to be tested and moving tests participants enacted a form of care that formed and maintained relationships. This at once modified and built their social field, and potentially changed their therapeutic outcomes. While migrants are usually seen as moving for obtaining care, the mobility of test results highlights that patients are not the only mobile element that impacts their care. When seeking to be considered and reach a diagnosis leading to effective treatment, participants who could resort to the private sector used tests to (1) submit further evidence to public practitioners in several cases, and (2) turned to private doctors with outcomes produced within the NHS in a few instances. For example, Dorota accessed private services within the UK. She complemented NHS-prescribed treatments with private tests to tackle the source of the severe headaches she experienced which made it difficult for her to work. When these did not result in the care she deemed appropriate within the NHS, she took these private test results to private Polish professionals in the UK:

I went to a GP; she listened and referred me for an MRI… It is a very expensive test [for the NHS]… I had to wait for it… The results didn't show anything. In the meantime, I had done a blood test privately, which suggested that there could be inflammation in the muscles. I showed the results to the doctor and she said it was completely unrelated, as there were no muscles in the head… That's interesting in itself -to a doctor say something like that… She concluded that since the MRI results hadn't shown anything, that meant there was nothing wrong with me. […] So I went to a Polish neurologist at the clinic in [name of place]; he looked at all the tests’ results and said that because the MRI scan was clear, it meant there was no tumour, no issue like that, and said that I had strained […] a group of muscles covering the head. He said that it was very common, prescribed me medication and I was fine two days later.

[Dorota, F, early-50s]

Having access to test results meant also that when interviewees were able to judge their health condition as not being properly addressed within the NHS, they could push to be considered by another health professional within the public health sector by accessing tests done privately, showing them within the NHS, and changing the care they received within NHS. This was the case for Iwona. After her GP did not consider it appropriate to refer her for tests given her symptoms, she decided to pursue them herself, and using the diagnostic evidence she gathered she convinced her GP to further investigate her disease:

I had a blood test done privately [in Poland]. The results showed that I had over 500,000 platelets in my blood. I repeated the test in Poland and the results were confirmed. After returning to the UK, I went back to my GP and showed him the results. At first, he said that it was just inflammation and that I was not to worry about it. I insisted, however, and was finally referred to a haematologist. After having a series of tests done, it turned out that it was blood cancer.

[Iwona, F, mid-40s]

Through multiple movements within, outside, and between sectors migrants changed their therapeutic trajectories. Crucially, these movements were initiated and managed by patients who sought care by moving to be tested and with tests and encountering different medical professionals. Notably, movements that build upon and modify social fields are not unequivocal and unidirectional.

## 4 Discussion and conclusion

Our results have been organized in three thematic strands which show the heterogeneity of practices and opinions of our participants while emphasizing the common ways in which tests are understood and used by them. Firstly, test results mediate migrants’ understanding of their health and of the healthcare they receive. While the satisfaction of our participants with the care they received varied, it was centred around common elements such as the willingness of doctors to refer them for tests and their availability to discuss results with them. Thus, the relationships of the participants with medical doctors were central in their undertaking of diagnostic procedures which in turn informed the quality of these relationships. Secondly, patients undertook movements motivated by the will to be tested. Participants varied in their movements, which were directed within the NHS and outside of it, and towards the private sector in the U.K. and especially in Poland. Crucially, these movements entailed the severing, creation of strengthening of relationships with medical professionals. Thus, the relationality involved in testing outlined above was multiplied and modified through patients’ movements. Crucially, we find that because most of these movements involve a degree of physical mobility and rely on material resources, they are unevenly accessible to our participants. Thirdly, participants not only moved to be tested but also moved test results between sectors and professionals. While the extent and trajectories of these movements varied, they relied upon and impacted relations with professionals and were not necessary unequivocally from or towards the public healthcare sector. We found that these movements had therapeutic effects on participants, who decided how to be treated depending on the encounters they sought with medical professionals. Below we expand on these findings by applying an analysis stressing the convergence of therapeutic mobilities, social fields, and medical encounters.

We have shown the double understanding mediated by medical tests: to understand one own's health and the care received. By deepening the analysis of what tests mean for Polish migrants in the UK, and how they are used, we have defined their salience in healthcare-seeking practices. When, during the medical encounter, the medical and individual narratives aligned (Jutel, 2009), patients were happy and proceeded with the treatment suggested. However, when they did not align, patients judged their care negatively. Crucially, while what leads the physician away from the ‘person-as-ill’ is usually the pursuit of objectivity through the use of technology (Leder, 1990), in our case, it is the patients who seek diagnostic testing. Further, participants sought to be tested and to have tests explained. Thus, rather than being confined to the list of the grievances of migrants’ healthcare service use, or commodity bought during medical returns, what we have found is that tests are a central component of transnational healthcare practices which implies the complexity of the relationality between patient and doctor. Both seeking to be tested and seeking to understand test results imply a clinical encounter. When samples and screenings become “technological texts” (Jutel, 2011 pg. 126), patients want to have access to them. Patients' understanding of their health is mediated by test results as they are interpreted and explained by physicians. Clinical encounters were both the occasion to ask for clarification and referral, and to receive an explanation of diagnostics.

Our findings confirm that diagnostic screening is a service sought transnationally and that diagnostic tests have certain characteristics that make them a desirable product (Main, 2016, Jackowska et al., 2012). However, the desire to have results at hand shows the significance of tests in enabling patient mobility. By focusing on diagnostic tests not only as a sought-after commodity, but also is implicated in various mobilities, we have shown that they are a means to establish and maintain relationships, and even when they are relatively accessible to lay persons they can facilitate productive dialogue between patients and doctors. Thus, tests are both a commodity acquired, or service sought, and a precondition to purchase or access service (i.e., the consultation) to obtain ‘appropriate’ treatment. We have considered how patients, tests, and clinicians come together during clinical encounters which were determinants for the creation of social fields within which patients sought care. By situating these encounters within a transnational social field and clarifying the roles of tests within them, we have exposed how patients acted upon their health through being involved in a series of mobilities. Through these mobilities, participants created, widened, and modified the social fields within which they managed their healthcare. Most evidently, diagnostic testing was accompanied by a specialist visit. Moreover, the sociality implicated in these mobilities became evident when they intersected with the tests results themselves. While tests are transformed into test results they become not only “readable”, but also detachable from patient and physician (Leder, 1990), and thus, we might add, they become portable. Our focus has been on the patient-doctor dyad within the wider social field. This focus has enabled the appreciation of the multiplication of possibilities by the creation or maintenance of relationships with doctors who are central in the delivery of care.

In taking diagnostic tests as a commodity and as an enabler of social fields, migrants emerge as creators of their own ‘therapeutic itineraries’ (Kangas, 2002) within often unequal conditions. By accessing test results our interviewees move between professionals operating across different countries. While the anxiety of not being diagnosed in the host country (Lee et al., 2010), and the cheaper and faster access to tests in the home country (Horton and Cole, 2011) have been found as characterizing the movement of migrants towards private services in the homeland, we have shown that this movement does not necessarily happen in this direction only and often involves accessing the private and public sectors of the country of settlement. Movement within transnational social fields changes direction and is possibly circular (Levitt and Glick-Schiller, 2004). This matches our findings which show that migrants move with test results not necessarily away from the national healthcare system in which they are originally embedded (Troccoli et al., 2021, Bochaton, 2015, Krause, 2008).

By understanding these mobilities as generative of therapeutics it is possible to appreciate their transformative potential (Kaspar et al., 2019). This is evident in the case of referrals and diagnoses prompted or modified by the movement of test results, in which patients’ circulating tests modify their social field, potentially creating a relationship with other professionals, who prescribe further tests, change diagnosis and recommend (or not) treatment which ultimately affects their health and how they feel. By coming together at specific times and places, tests, patients and clinicians prompt the transformation of therapeutic itineraries and new mobilities with therapeutic effects. Thus, the mobilities we have analysed are transformative of patient-migrants’ bodies and understanding of themselves. When patients create, maintain, and modify a transnational social field, it can have transformative repercussions on the care they receive from institutions, such as the NHS, in which they are embedded and on the development of the private sectors to which they reached out to receive care from. In analysing the intersections between mobilities of patients and tests within a social field approach we have contributed to the understanding that patients are not atomized (Bell, Holliday, Ormond, and Mainil, 2015), but actively partake in the processes in which they are embedded and in which they operate with other actors.

While we have stressed the mobility and connectivity between patients, tests and physicians, they are not unpatroned or omnidirectional. On the contrary, such relationships are uneven. We have shown how access to tests and consultations is contingent on the availability of time and resources which are not available to all our interviewees. Participants who are able and willing to seek healthcare beyond the national healthcare system to seek a diagnosis or monitor a specific health condition do so within transnational social fields that are more or less reliant on actors located within the private sector. This is because consultations, tests and treatments are contingent on each other but also distinct and defined. The unequal exchange, organization, and transformation of resources are key to understanding social fields (Levitt and Glick-Schiller, 2004). Thus, beyond the fact that access to healthcare transnationally is itself unequal, how distinct elements of therapeutic itineraries are sourced within the transnational social field by migrants also emerges as being fraught with asymmetries.

This study is the first to provide detailed insights on the understanding and use of diagnostic testing in the engagement with healthcare practices by Polish migrants in the U.K. beyond the impact of specific screening programmes. While the interviews were conducted in 2020 we believe that the findings remain relevant since the Polish population in the U.K. has remained stable at the time of writing and the findings reflect broader patterns of healthcare utilization highlighted in the literature of the last twenty years. More broadly, the article contributes to the literatures on migrants’ healthcare and transnational practices by showing the pivotal role of tests in the creation and modification of social relationships by which migrants manage their health. By applying to diagnostics the questions of who and what moves in transnational healthcare it shows the complexities of practices that result in therapeutic outcomes. Thus, the article suggests the need to further investigate the role of diagnostics in migrants’ healthcare practices and calls for future research to consider testing as a central component of these practices. This study has also a number of limitations in relation to the sampling and advertisement strategy. Women are overrepresented in our sample (69%) and participants are all residing in England. Further, most of the participants have been living in the U.K. between 5 and 14 years (78%), while a minority have resided there less than 4 (13%) or more than 15 years (9%). Thus, it is important that future research assesses these practices for Poles living in the other three countries of the U.K. and with more than 15 years of residency.

Finally, the results of this study provide valuable insights for policymakers. The availability of ad hoc and regular testing and of test results impacts patients’ understanding of their health and the care they receive. It influences patients’ ability to make informed choices about their therapies, to decide if to seek multiple medical opinions, and to repeat or undergo further diagnostic procedures. Therefore, patients should be clearly informed about the rationalities behind medical decisions regarding diagnostic testing, be able to easily access their test results and discuss them thoroughly with the physicians who assist them. As well as facilitating the speediness of therapies, this has the potential of enhancing patients’ understanding of medical procedures and strengthening the patient-doctor relationship through the building of trust pivotal for patients’ ongoing and future health outcomes, and their adherence to therapeutic recommendations.

## Data availability statement

The data that support the findings of this study are not available due to their confidential nature.

## Funding statement

This work was supported by the ESRC under Grant number 518928101, Centre for Population Change Transition Funding.

## Ethics approval and participant consent statement

The Research Integrity and Governance team and the Faculty of Social Sciences Ethics Committee at the University of Southampton have granted full ethical approval (52861/52861.A1). Informed consent was given by survey participants at the beginning of the online questionnaire and by interviewees via filled forms returned electronically.

## CRediT authorship contribution statement

**Giuseppe Troccoli:** Conceptualization, Data curation, Formal analysis, Investigation, Methodology, Writing – original draft, Writing – review & editing. **Chris Moreh:** Conceptualization, Methodology, Supervision, Writing – review & editing. **Derek McGhee:** Conceptualization, Funding acquisition, Methodology, Supervision, Writing – review & editing. **Athina Vlachantoni:** Conceptualization, Funding acquisition, Methodology, Supervision, Writing – review & editing.

## Declaration of Competing Interest

The authors declare that they have no known competing financial interests or personal relationships that could have appeared to influence the work reported in this paper.

## Figures and Tables

**Table 2:** Interview guide for participants with a chronic/long term condition or disability.

1	What healthcare services do you usually access to manage your condition? Apart from healthcare services, are there any other services that you access in relation to your condition?
2	Who are the professionals whom you usually see with regards to your condition?
3	During your time in the UK, have you changed the kind of services or the professionals that you access?
4	Has your access to the services you currently use changed due to the current COVID-19 (Coronavirus) outbreak?
5	Did you know you had/Have you had this condition before coming to the UK? [If Yes] Can you tell me about the circumstances of your diagnosis [in Poland]? How did you manage to have your diagnosis accepted in the UK? Were you diagnosed again in the UK? What were your thoughts about your condition when you decided to move to the UK? How important was moving to the UK from the perspective of caring for your condition? [If No] Can you tell me about the circumstances of your diagnosis in the UK?
6	Apart from healthcare professionals, does anyone assist/help you with your condition or with any aspects of your life related to your condition?
7	Does the current Coronavirus outbreak have an impact on the way in which you are assisted?
8	Do you provide assistance to anyone?
9	Have you ever thought of moving back to Poland because of your condition?
10	[If diagnosed in Poland] What are the differences/similarities in terms of being treated for your condition in Poland and the UK? What are the differences in terms of receiving assistance from other people who are not healthcare professionals?
11	[If diagnosed in the UK] What do you think would be different/similar if you were treated in Poland? What would be different in terms of receiving assistance from other people who are not healthcare professionals?
12	Thinking of how you care for yourself and the care you receive from others, are there any advantages/disadvantages in being in the UK rather than Poland?
13	How does your condition impact your travels to Poland?
14	Do you access/have you accessed any services in relation to your condition during your visits to Poland?
15	Have your plans to travel to Poland changed because of COVID-19?
16	What is your current occupation? What was your occupation in Poland before moving to the UK? What occupations did you have while in the UK?
17	[if currently working] How do you manage taking care of your condition in relation to your work? [if currently not working] What is the relationship between your current employment status and your condition?
18	How are the recent Coronavirus-related changes impacting the way in which you work and manage your condition?
19	How do you think that Brexit will change any aspect of your care?
20	Would you like to say something about any of these aspects that we haven't discussed or highlight something that we have already talked about?

**Table 3:** Interview guide for participants caring for someone with a chronic/long term condition or disability (not in a professional capacity).

1	Could you describe the ways in which you take care of the CR (Care receiver)?
2	Do you also provide care to someone else?
3	What is the significance for you of caring for the CR?
4	What is your current occupation? What was your occupation before moving to the UK? Have you changed occupation since you moved to the UK?
5	[if working] How do you manage taking care of this person in relation to your work?
6	[if not working] Do you think that your current employment status is related to the care you provide?
7	How are the recent Coronavirus-related changes impacting the way in which you work [your partner or another carer] and manage caring?
8	Is someone assisting you in caring for this person? [if Yes] What are the ways in which they are assisting you in caring for this person? [if CR in Poland] How do you stay in touch with them? Did you change the way/frequency/content of your contacts [with the other carer] since the start of the COVID19 pandemic?
9	[if CR in UK] Are you assisting the CR in accessing any services?
10	[if CR in PL] What services does the CR access in Poland? Do you help the CR in accessing these services?
11	Did the CR ever come to the UK to access services?
12	How is your capacity to help the CR in accessing these services changing in relation to the COVID-19 (Coronavirus) outbreak?
13	[if CR UK] Do you travel to Poland with the CR? [If Yes]Do you access services there?Who do you visit when there?Do your parents and siblings help you to care for the CR while you are there?
14	[if CR in PL] When you travel to Poland, do you stay with the CR? What do you do together when you are there?[If kin are co-carers]: How is your relationship with your kin while you are in Poland? What do your siblings do differently when you are there?
15	Do you think that Brexit will impact the use of these services? Are you thinking of arranging things differently in the future because of Brexit?
16	Have your plans to travel to Poland changed because of COVID-19?
17	[if CR Poland] How do you stay in touch with CR?Has the frequency, content, and ways of communication changed since the start of the pandemic?
18	Did the CR need care when you were in Poland?[If Yes] a) What were your thoughts about the CR when you decided to move to the UK? b) How important was moving to the UK for taking care of the CR?[if No] How would be different to care for this person if you were in Poland now?
19	Were you caring for this person while in Poland? [If Yes] What are the differences and similarities in caring for the CR between when you were in Poland and now?What services did you use to access in Poland with the CR?
20	[if CR UK] Are there any advantages/disadvantages for you in being in the UK with the CR.
21	[if CR in PL] Are there any advantages/disadvantages in being in the UK rather than Poland with the CR?
22	[if CR in PL] Do you help materially the CR?
23	Would you like to say something about any of these aspects that we haven't discussed or highlighted something that we have already talked about?
24	How do you think that Brexit will change aspects of your care?

## Appendix A

The tables below summarize the interview guides used for this project. When participants identified with both categories covered in Tables 2 and 3, they were asked both sets of questions. Note that the order of the questions changed according to the circumstances of the interview and the themes brought forward by the interviewees.
